# Liquid-Phase Synthesis of Cyanuric Acid from Urea

**DOI:** 10.3390/molecules15031898

**Published:** 2010-03-16

**Authors:** Dong-Mei She, Hai-Lin Yu, Qi-Liang Huang, Fen-Ming Li, Chun-Jiu Li

**Affiliations:** 1 College of Science, China Agricultural University, Beijing 100193, China; E-Mail: cjiuli@cau.edu.com (C-J.L.); 2 Plant Protection Institute of the Chinese Academy of Agricultural Sciences, Beijing 100193, China; E-Mails: yuhaili6258@163.com (H.-L.Y.); qlhuang@ippcaas.cn (Q.-L.H.)

**Keywords:** cyanuric acid, urea, liquid-phase, yield

## Abstract

The focus of this paper was to identify a cheaper solvent from among diesel fuel, kerosene, sulfolane or a mixture of sulfolane and cyclohexanol for the preparation of cyanuric acid heterocyclization of urea. To obtain a higher yield, the effects of catalyst (sodium, ammonium, calcium and zinc salts) and temperature (160 °C to 220 °C) on the trimerization of urea were also carefully studied. We established the optimal reaction conditions and further validated them in our scale-up experiments.

## Introduction 

Cyanuric acid (2,4,6-trichloro-1,3,5-triazine or isocyanuric acid) is a key intermediate for the synthesis of a variety of organic compounds, include epoxy resins, chlorinated derivatives, detergents, antioxidants, dyes, pesticides, antitumour agents and so on. It is also the raw material for many new products such as trichloroiminocyanuric acid (TCCA), 1,3,5-tri-2–propenyl-1,3,5–triazine-2,4,6-(1*H*,3*H*,5*H*)-trione (TAIC), triglycidyl isocyanurate (TGIC) and tricarboxylethyl isocyanurate (TCIC). These new products are well known and widely used in chemical engineering, light manufacturing, electrical appliances, mechanical industry, spinning and agriculture [1,2,3,4].

Cyanuric acid is a white crystalline solid, with a melting point of 145–148 °C and a boiling point of 190 °C. Various methods have been reported for the synthesis of cyanuric acid. Those methods can be divided into two types. One involves production of cyanuric acid from urea as starting material and the other involves polymerization of cyanogen chloride, or treatment of HCN and Cl in the presence of a catalyst [5]. Of the two routes, the use of urea is more traditional, economical and convenient as one can typically obtain higher quality products in better yield. Moreover, it is easier to scale-up for manufacturing with ordinary equipment. 

There are two ways for preparing cyanuric acid from urea. One is by heating urea at high temperature (≥ 250 °C) without solvent, and the other is the trimerization of urea in a solvent, with or without a catalyst [6,7,8,9]. The first method has the disadvantage that since the reaction product changes from a solid phase to a liquid phase and back again to the solid phase, so it may be agglutinated or solidified and adhere to the heating surface or appropriate heat transfer surface, with the result that the product quality is lowered and the reaction equipment can be damaged [4]. At the same time, the product can’t be used directly in the chemical industry without refining. Comparatively, the second method is environmentally benign, safe, low cost and the product can be used unrefined, but this method has one weak point, in the high boiling point solvent must be regenerated after being used a few times, and some of the potentially expensive solvents used like sulfolane, tetraethylene glycol dimethyl ether, butyl carbitol and cyclohexanol may be wasted.

**Scheme 1:**
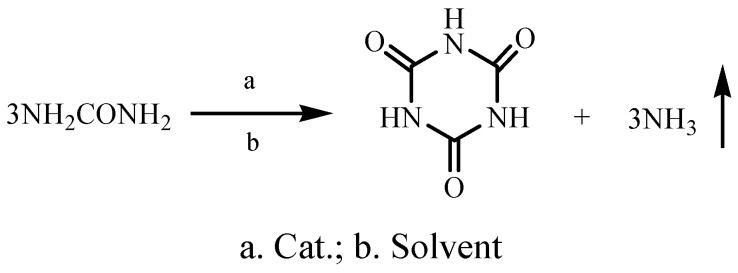
The synthesis of cyanuric acid.

In our study, the procedure for the trimerization of urea in a solvent was further developed (Scheme 1). We tried to identify a cheaper solvent like diesel or kerosene for urea trimerization and cyanuric acid was obtained from those high boiling point solutions. To our attempts to improve the yield by investigation of the influence of catalyst, we obtained different results under our study condition from those obtained by Kawahara and Tejima [7] in which cyanuric acid was prepared by heating urea in the presence of alkali metal hydrides, alkali metal amides, and alkali metal salts in organic solvents. For example, urea was treated with NaH in tetraethylene glycol methyl ether at 150 °C for 5 hours to give 99% cyanuric acid, whereas 0% cyanuric acid was obtained without NaH [7]. In our study, the catalysts we have selected for the reaction did not improve the yield much. The influence of temperature on the result was also carefully studied, and the optimal reaction conditions established were validated in our subsequent larger scale experiments.

## Results and Discussion

Considering that the trimerization of urea should take place at a high temperature [11,12], by heating urea or biuret in an inert solvent (sulfolane, 4-chloro-*m*-cresol, 3-methylsulfolane, 2-methoxyethyl ether), and better at temperatures above 180 °C [11], our initial efforts focused on the solvent to be used. Three kinds of single solvent: kerosene (low odor paraffinic solvent, CAS number 64742-47-8, boiling point 220~270 °C), diesel (light diesel oil, CAS number 68334-30-5, boiling point 250~400 °C) and sulfolane, and one mixture of solvents - sulfolane and cyclohexanol - were tested first (Table 1). Cyanuric acid was obtained in a good yield (88.7% and 80.5%, respectively) in the single solvents kerosene and diesel at 180 °C (Table 1). Maybe it was propitious to form the cyanuric acid in a solvent of lower polarity, so the nonpolar solvents kerosene and diesel are better than the polar solvents sulfolane and cyclohexanol. Kerosene and diesel are also cheap and can probably be recycled.

Next we examined the effects of the temperature of reaction (Table 2). The experimental results show that the highest yield (88.9.0%) was obtained at 190 °C. The temperatures from 180 °C to 210 °C did produce different yields of cyanuric acid, but the reaction completed faster at 210 °C. The process appeared to be accelerated at higher temperatures while the yield did not increase correspondingly. At reaction temperatures higher than 210 °C, urea could be lost when the reaction was under vacuum, and the yield was decreased. To confirm this a cold trap was placed between the reactor and the pump and white urea was found in the trap. An interesting phenomenon was observed in that the solution appeared turbid at the beginning of the reaction process due to the presence of undissolved starting materials, while the reaction mixture gradually became clear in the middle of the reaction time and turbid again in the end as a result of the appearance of the insoluble product.

In our research the yield changed a little with the catalyst species tested (Table 3). The highest yield (82.4%) was obtained with (NH_4_)_2_SO_4_. Different catalysts resulted in small changes in yield. This result differs for that seen when urea is heated directly without solvent, in which many kinds of salts can accelerate the reactions and increase the cyanuric acid yield. In our experiments when cyanuric acid is prepared in an organic solvent, the catalyst does not promote the yield of cyanuric acid much nor accelerate the reaction either. Perhaps the salts make the system melt at a lower temperature and facilitate cyclization of the urea in the solid state reaction without solvent. There are maybe other reasons for the negligible influence of the tested catalysts on cyanuric acid yield, so this needs further exploration.

## Experimental

### General

All chemicals were of reagent grade and used as purchased. ^1^H-NMR (300 MHz) and ^13^C-NMR (75 MHz) spectra were recorded on a Bruker AC-P300 spectrometer using CDCl_3_ as the solvent; TMS was the internal standard. IR spectra were determined on an FTS-185 instrument as neat films. All reactions were carried out in pre-dried glassware (150 °C, 4 h) cooled under a stream of dry air. 

### Synthesis of cyanuric acid

The synthesis of cyanuric acid is depicted in Scheme 1. Urea (20 g) with (or without) catalyst was added to solvent (40 mL) in a round-bottom flask. The mixture was stirred at 150 °C, placed under vacuum (10 mm Hg) and then heated at 160~220 °C. The reaction was monitored by pH test paper to test emission of NH_3._.When the pH test paper did not change color anymore the reaction was considered complete. The reaction mixture was cooled to 80 °C, put into water (15 mL) and stirred for 1 hour to precipitate the product. The obtained dry solid was heated to 150 °C for 2 hours to remove the crystallization water and afford pure cyanuric acid. The structure of the product was confirmed by m.p., IR, ^1^H-NMR and ^13^C-NMR spectra.

## Conclusions 

In conclusion, liquid-phase synthesis of cyanuric acid from urea in a stable high yield, convenient operation and low cost, is possible using kerosene as solvent.

## Figures and Tables

**Table 1:** Effect of different kinds of solvent on experimental results.

Entry	Solvent	Yield (%)
1	kerosene	88.7
2	diesel	80.5
3	sulfolane	54.8
4	sulfolane: cyclohexanol = 3:1	64.6

*Conditions*: 180 °C, solvent (v)/urea (q) = 2:1.

**Table 2:** Effect of reaction temperature on experimental results.

Entry	Temperature (°C)	Yield (%)
1	180	85.7
2	190	88.9
3	200	87.4
5	210	80.4
5	220	67.5

*Conditions*: kerosene(v)/urea (q) = 2:1.

**Table 3:** Effect of catalyst on experimental results.

Entry	Catalyst	Yield (%)
1	(NH_4_)_2_SO_4_	82.4
2	CaCl_2_	77.6
3	NH_4_Cl	80.9
4	ZnCl_2_	79.4
5	KNO_3_	82.4
6	NaCl	76.3
7	NH_4_NO_3_	80.6
8	CaSO_4_	75.3
9	Ca(NO_3_)_2_	77.3
10	ZnSO_4_	81.2
11	ZnNO_3_	82.0

*Conditions*: kerosene, solvent (v)/urea (q) = 2:1; temperature: 190 °C.
